# Prevalence of Thiamine Deficiency in Pregnancy and its impact on fetal outcome in an area endemic for thiamine deficiency

**DOI:** 10.1371/journal.pntd.0011324

**Published:** 2023-05-30

**Authors:** Ozaifa Kareem, Samiya Mufti, Sobia Nisar, Masood Tanvir, Umar Muzaffer, Nahida Ali, Ishfaq Ahmad Sheikh, Ghulam Nabi Bader

**Affiliations:** 1 Department of Pharmaceutical Sciences, University of Kashmir, Srinagar, India; 2 Department of Gynecology and Obstetrics, Government Medical College, Wazir Bagh, India; 3 Department of Medicine, Government Medical College, Srinagar, India; University of Washington, UNITED STATES

## Abstract

**Background:**

Pregnancy is a metabolically challenging state with increased nutritional demand. Thiamine is an important cofactor in various metabolic pathways and thus its deficiency could have a serious impact on both maternal and fetal outcomes. Kashmir has thiamine deficiency in endemic proportions, with multiple reports of infantile beriberi, postpartum neuropathy, and gastric beriberi. This prompted us to assess the extent of the burden of thiamine deficiency during pregnancy.

**Methods:**

This cross-sectional study was conducted for a period of two years in pregnant women attending the antenatal clinic. A demographic, clinical, biochemical, and dietary assessment was done in all participants. The whole blood thiamine levels were assessed by high-performance liquid chromatography.

**Results:**

A total of 492 participants were included in the study with a mean age of 30.30±4.57 years and a mean BMI of 24.25±3.32 Kg/m2. The mean whole blood thiamine level of all participants was 133.29±14.32 nmol/L. Low thiamine status was present in 38.2% (n = 188) of participants. Participants with low thiamine had poor perinatal outcomes, with 3.1% (n = 6) reporting early infant death.

**Conclusion:**

A high prevalence of thiamine deficiency occurs in pregnant women of Kashmir. Low thiamine is associated with poor nutritional status as well as poor perinatal outcomes.

**Trial registration:**

CTRI/2022/07/044217.

## Author summary

Thiamine is an essential micronutrient with a pivotal role in mitochondrial energetics and oxidative and nonoxidative carbohydrate metabolism. Thiamine deficiency is a frequently neglected problem of significance across Southeast Asia, where beriberi is endemic. There are multiple reports of infantile beriberi, gastric beriberi, high-output heart failure and postpartum neuropathy from Kashmir, a northern Indian state. The reports of infantile beriberi in exclusively breastfed infants suggest maternal thiamine deficiency during pregnancy. Additionally, pregnancy puts a physiological strain on micronutrient physiology to fulfil the demands of the mother, placenta, and fetus. Although recent studies have reported thiamine deficiency-related diseases in the region, this is the first representative data assessing the biochemical evidence of thiamine deficiency in pregnant women from Kashmir. We conducted a cross-sectional explorative study to describe the prevalence of thiamine deficiency in pregnant women of Kashmir and its perinatal outcome. A total of 492 women across three trimesters of pregnancy were enrolled for the study. We found a high prevalence (38.2%) of thiamine deficiency among pregnant women of Kashmir. The low thiamine status was associated with poor socioeconomic status, poor nutritional status and higher consumption of staple polished white rice, and tea. Our study also reported poor perinatal outcome in association with the low thiamine status of the mother, suggesting a possibility of thiamine deficiency in the early neonatal period.

## Introduction

Vitamin B1, also known as thiamine, is a water-soluble vitamin that is pivotal in several metabolic pathways [1]. Thiamine diphosphate (ThDP), an active form of thiamine, is utilized by the major enzyme complexes involved in carbohydrate metabolism (for energy production by ATP synthesis), lipid metabolism (for production and maintenance of myelin sheath in the brain), and the production of amino acids and glucose-derived neurotransmitters (e.g., glutamic acid; GABA) [2]. Since thiamine is not synthesized endogenously and has a short half-life (1–12 hr), an adequate dietary supply is needed to maintain tissue thiamine levels [3]. Deficiency of this vitamin leads to various cardiovascular and neurological consequences in adults [4], collectively known as ‘beriberi’[5]. Classically, cardiovascular sequelae are known as wet beriberi while neurological features are referred to as dry beriberi [3]. While being nearly eradicated elsewhere, thiamine deficiency remains a public health concern in many parts of the world, especially in Southeast Asia [6].

Pregnancy places a unique physiological strain on micronutrient physiology in order to fulfil the demands of the mother, placenta, and fetus [7]. Newborns are at an increased danger of developing thiamine deficiency if the thiamine levels of their mothers are inadequate during pregnancy and/or lactation [8]. It has previously been reported that the fetus receives thiamine preferentially through the placental transfer at the expense of the mother [9]. However, lactating women with subclinical thiamine deficiency secrete a suboptimal amount of thiamine in breast milk, predisposing the infants to overt infantile beriberi [10]. It is interesting to note that it is not uncommon for infants to show symptoms of beriberi while their mothers continue to show no signs of the disease [11,12]. New evidence suggests that infants with milder forms of thiamine deficiency, those not severe enough to cause beriberi, can still be capable of causing cognitive impairments later in life [13]. Subclinical thiamine deficiency in pregnant mothers has also been reported to adversely affect the balance, fine motor skills and language development in preschool children [13,14].

Recently, a number of reports of infantile beriberi in exclusively breastfed infants have been reported from Kashmir, [15–18] suggesting maternal thiamine deficiency during pregnancy. This has essentially been related to a high carbohydrate diet (in the form of rice) poor in thiamine [19]. The peculiar regional dietary habits along with the postpartum dietary restrictions have likely been implicated as a cause of thiamine deficiency in this region [20–22]. Barring a few reports of postpartum polyneuropathy due to thiamine deficiency [23], there is no data available to substantiate the thiamine status or the burden of thiamine deficiency in pregnant women of Kashmir or its impact on neonatal outcomes. In view of the various reported cases of infantile beriberi, and the possibility of subclinical deficiency affecting neurocognitive development, we suspect a high prevalence of thiamine deficiency in pregnancy. Herein, we report the clinical characteristics, laboratory findings, and whole-blood thiamine levels in pregnant women of Kashmir valley.

## Participants and methods

### Ethics statement

The ethical approval for the study was obtained from the Institutional Ethical Committee, Government Medical College, Srinagar vide No: 255/ETH/GMC/ICMR dated 14/4/2021. A well–informed written consent was taken from all the study participants. The procedures followed were in accordance with the Helsinki Declaration.

This cross-sectional explorative study was conducted in Government Medical College and associated hospitals for two years, from November 2020 to October 2022. Pregnant women, across all three trimesters, attending the antenatal clinic were enrolled. The study was registered with the Clinical Trial Registry of India under CTRI registration no. CTRI/2022/07/044217. Eligibility criteria included all the pregnant women attending the antenatal clinic while those who did not provide consent or were taking thiamine-containing supplements were excluded. A total of 567 were screened, of which 27 refused to participate, 19 refused to give a blood sample, 8 had a history of multivitamin intake, 11 were lactating and 10 had a complicated pregnancy. A detailed standardized study questionnaire was administered to all the participants and information regarding demographics, anthropometry, and clinical assessment was done. The data regarding socioeconomic status was also taken. A detailed dietary pattern was obtained by employing a Food Frequency Questionnaire (FFQ) and 24-h dietary recall to assess the various diet components using diet software that was specifically built for this purpose (Diet Cal, Profound Tech Solutions, New Delhi, India). The infant mortality was estimated by verbal autopsy at the time of follow-up of patients.

A 5 ml venous blood was collected from all the participants by phlebotomy in VACUTECH K3-EDTA-containing tubes and VACUTECH gel+clot activator tubes (*Vacuette*, *Greiner Bio One*, *Austria*). Samples were transferred immediately to the central facility within 4 hours of collection. The samples were separated into 500 μL aliquots and frozen at -20°C and were batch-transported every day on dry ice for whole-blood thiamine analysis. Whole blood thiamine analysis was done using a modified method of Körner et al. 2009 [24]. Aliquots of blood samples were briefly thawed and treated with 400 μL of 1.5 M trichloroacetic acid to precipitate proteins and subsequent centrifugation. The prepared sample was run through ultra-high performance liquid chromatography (HPLC) (*Agilent model 1260 system*, *Infinity II*, *Singapore*) with a fluorescence detector (*Agilent model G7121A*, *Agilent Technologies*, *Singapore*) and an autosampler (*Agilent model G4294B*, *Agilent Technologies*, *Singapore)* to allow for online pre-column derivatization with potassium ferricyanide. Coats and colleagues [25] have employed a reference range of whole-blood thiamine levels of 70–180 nmol/L among self-reported healthy American adults. Similarly, based on values from 68 healthy Dutch blood donors and laboratory staff aged 20–50 years the Institute of Medicine (IOM) [26] has defined thiamine deficiency as whole-blood thiamine levels of <70 nmol/L and marginal deficiency as 70–90 nmol/L. Although there are no universally agreed-upon cut-offs for thiamine status as measured by ThDP [27], however, a whole-blood ThDP cut-off of <74 nmol/L corresponding to 2.5 μg/dL was employed in our study, which aligns with the reference interval set by Lu and Frank [28]. Based on this, our study population was divided into two groups, normal thiamine, and low thiamine group.

The statistical analysis was conducted using SPSS version 26.0 (IBM, Armonk, NY, USA). Data was evaluated for normal distribution as per Kolmogorov–Smirnov test. Continuous variables were expressed as mean ± SD. Comparison for continuous variables was estimated by Mann–Whitney U test (parametric variables) and Kruskal-Wallis test (non-parametric variables) and categorical data were compared by χ2 test. For comparison of demographic and biochemical parameters between the normal thiamine and low thiamine group, we used paired independent simple t-test. For all comparisons p-value ≤0.05 was considered significant.

## Results

A total of 492 participants were enrolled for the study. The demographic and biochemical characteristics of the study participants are given in Table 1. The average age of the study population was 30.30±4.57 years. The mean BMI of the population was 24.25±3.32 Kg/m^2^. Among all, 8.4% (n = 42) were in the first trimester, 22.4% (n = 113) were in the second trimester, and 67% (n = 337) were in the third trimester of pregnancy (Table 2). The mean whole blood thiamine level of all participants was 133.29±14.32 nmol/L. The mean thiamine of the participants registered in the first trimester was 141.52±5.88 nmol/L. Those belonging to the second trimester had a mean thiamine level of 133.75±3.91 nmol/L, and those in the third trimester had an average whole blood thiamine of 130.41±4.98 nmol/L. Low thiamine status was present in 38.2% (n = 188) while 61.7% (n = 304) had normal thiamine levels. Nearly 59.3% of participants (n = 292) belonged to the lower-middle socioeconomic status of which the majority of participants 66.5% (n = 125) belonged to the low-thiamine group while only 54.9% (n = 167) belonged to the normal thiamine group (p = 0.031).

**Table 1:** Demographic and biochemical parameters of the study population (n = 492).

Parameter	All Participants (Mean±S.D)	Low thiamine (Mean±S.D)	Normal thiamine (Mean±S.D)	P-value
Age (years)	30.30±4.57	30.08±4.740	30.44±4.46	0.397
BMI (kg/m^2^)	24.25±3.32	24.26±3.29	24.25±3.34	0.616
Systolic blood pressure, mmHg	123.24±14.50	124.34±13.59	122.56±15.02	0.182
Diastolic blood pressure, mmHg	79.83±11.30	80.52±10.90	79.41±11.53	0.284
Pulse (beats/min)	90.45±12.91	91.41±13.08	89.85±12.79	0.188
Serum urea, mg/dL	26.32±6.53	25.15±6.34	27.81±6.88	0.024
Serum creatinine, mg/dL	0.88±0.21	0.87±0.15	0.85±0.17	0.118
Serum bilirubin, mg/dL	0.58±0.47	0.56±0.30	0.61±0.47	0.283
Serum total protein, g/dL	7.28±0.75	6.99±0.71	7.32±0.69	0.037
Serum albumin, g/dL	4.20±0.61	3.89±0.63	4.33±0.60	0.029
Uric acid, mg/dL	4.55±1.26	4.76±1.26	4.75±1.28	0.890
Serum glutamic oxaloacetic transaminase, IU/L	34.24±10.98	30.46±10.64	30.43±22.45	0.287
Serum glutamic pyruvic transaminase, IU/L	31.06±25.38	30.22±22.73	32.52±26.29	0.850
Serum alkaline phosphatase, IU/L	103.40±7.14	99.15±28.29	106.02±27.39	0.195
Serum sodium, mEq/L	136.67±7.30	136.63±7.06	136.69±7.4	0.931
Serum potassium, mEq/L	4.21±1.80	4.32±3.03	4.14±2.65	0.469
Blood glucose random, mg/dL	95.30±21.40	93.80±18.07	97.74±25.77	0.047
TSH	5.17±3.34	5.34±3.23	5.06±3.41	0.369
Hemoglobin g/dL	9.76±1.36	8.81±1.40	10.22±1.34	0.038
Neutrophil count *10^3^/mL	66.93±13.71	64.95±18.15	67.58±12.11	0.583
Red Blood cell count *10^3^/mL	3.95±0.57	3.87±0.55	4.12±0.59	0.028
Lymphocyte count %	27.22±12.01	30.41±13.13	25.82±11.30	0.071
White blood cell count, *10^9^/L	8.78±6.11	10.02±10.13	8.53±3.00	0.229
Blood thiamine diphosphate, nmol/L	133.29±14.32	46.25±18.10	187.12±115.61	0.001

**Table 2:** Characteristics of the study population (n = 492).

Characteristic	All Participants (n = 492)	Low thiamine Group (n = 188)	Normal thiamine Group (n = 304)	P-value
**SE Status**				
Lower	27 (5.4)	10 (5.3)	17 (5.6)	0.031
Upper lower	160 (32.5)	50 (26.6)	110 (36.2)	
Lower middle	292 (59.3)	125 (66.5)	167 (54.9)	
Upper middle	14 (2.8)	3 (1.6)	10 (3.3)	
**Gravida**				
1	222 (45.2)	85 (45.2)	137 (45.0)	0.488
2	122 (24.8)	41 (21.9)	81 (26.7)	
3	87 (17.7)	40 (21.2)	47 (15.5)	
>3	61 (12.3)	22 (11.7)	39 (12.8)	
**Para**				
0	223 (45.3)	85 (45.2)	138 (45.4)	0.703
1	140 (28.5)	49 (26.1)	91 (30.0)	
2	85(17.3)	37 (19.6)	48 (15.7)	
>2	44 (8.9)	17 (9.1)	27 (8.9)	
**Trimester**				
First	42 (8.6)	17 (9.1)	25 (8.2)	0.167
Second	113 (22.9)	39 (20.7)	74 (24.3)	
Third	337 (68.5)	132 (70.2)	205 (67.5)	
**Loss of appetite**				
First trimester	195 (38.8)	78 (41.4)	117 (38.4)	0.036
Second trimester	11 (2.2)	10 (5.3)	1 (0.3)	
Third trimester	8 (1.6)	7 (3.7)	1 (0.3)	
1&2 trimester	69 (13.7)	35 (18.6)	34 (11.2)	
All trimesters	13 (2.5)	3 (1.5)	10 (3.3)	
**Nausea during pregnancy**				
First trimester	251 (49.9)	97 (51.5)	154 (50.6)	0.047
Second trimester	7 (1.4)	5 (2.6)	2 (0.6)	
Third trimester	6 (1.2)	5 (2.6)	1 (0.3)	
1&2 trimester	78 (15.5)	37 (19.6)	41 (13.5)	
All trimesters	26 (5.2)	11 (5.8)	15 (4.9)	
**Vomiting During Pregnancy**				
First trimester	214 (42.5)	84 (44.6)	130 (42.7)	0.05
Second trimester	13 (2.5)	12 (6.3)	1 (0.3)	
Third trimester	9 (1.7)	7 (3.7)	2 (0.6)	
1&2 trimester	70 (13.9)	27 (14.3)	43 (14.1)	
All trimesters	21 (4.1)	9 (4.7)	12 (3.9)	
**Number of Retching Episodes Per Day**				
1	198 (39.4)	81 (43.1)	117 (38.4)	0.036
2	17 (3.4)	7 (3.7)	10 (3.2)	
>2	33 (6.5)	25 (13.2)	8 (2.6)	
**Weight loss**	2(0.4)	0 (0.0)	2 (0.6)	0.277
**Orthostatic hypotension**	12(2.4)	3 (1.6)	9 (2.9)	0.569
**Heart rate >100 bpm**	46 (9.2)	27 (14.3)	19 (6.2)	0.032
**Dry Skin**	1(0.2)	1 (0.5)	0 (0)	0.471
**Mood Changes**	19 (3.9)	4 (2.1)	15 (4.9)	0.187
**Lethargy**	62 (12.4)	33 (17.5)	29 (9.5)	0.001
**PIH**	45 (9.1)	19 (10.1)	26 (8.5)	0.563
**GDM**	36 (7.3)	12 (6.3)	24 (7.8)	0.442
**Anaemia**	17 (3.4)	12 (6.3)	5 (1.6)	0.016
**Dysuria**	28 (5.6)	16 (8.5)	12 (3.9)	0.085
**PND/Orthopnoea**	24 (4.8)	17 (9.1)	7 (2.3)	0.042
**Type of rice**				
Kashmiri	198 (40.2)	47 (25.1)	151 (49.6)	0.004
Ration	254 (51.6)	114 (60.6)	140 (46.1)	
Both	40 (8.2)	27 (14.3)	13 (4.3)	
**Storage**				
Bins	396 (80.5)	111 (59.1)	285 (93.8)	0.212
Kutch	96 (19.5)	77 (40.9)	19 (6.2)	
**Storage of grain**				
Same year	445 (90.4)	150 (79.7)	295 (97.1)	0.231
One Year	32 (6.5)	28 (14.9)	4 (1.3)	
Two year	15 (3.1)	10 (5.4)	5 (1.6)	
**Preparation of rice**				
One rice wash	97 (19.8)	1 (0.5)	96 (31.6)	0.021
Two rice washes	173 (35.1)	61 (32.5)	112 (36.8)	
Three rice washes	190 (38.6)	106 (56.4)	84 (27.7)	
Four rice washes	32 (6.5)	20 (10.6)	12 (3.9)	
**Discarding cooking water after boiling rice**	292 (59.3)	136 (72.3)	156 (51.3)	0.051
**Green leafy vegetables**	456 (92.6)	180 (95.7)	276 (90.7)	0.241
**No. of cups of tea per day**				
1	78 (15.8)	8 (4.3)	70 (23.1)	0.071
2	237 (48.1)	15 (7.9)	222 (73.1)	
>2	177 (35.9)	165 (87.8)	12 (3.8)	
**Intake of Pickle**				
Occasional intake	278 (56.5)	91 (48.5)	187 (61.5)	0.031
Daily intake	45 (9.1)	28 (14.9)	17 (5.6)	

The anthropometric parameters were comparable among the two groups while some biochemical parameters were significantly different. The mean serum urea of the participants was 26.32±6.53 mg/dL which was significantly lower in the low-thiamine group (25.15±6.34 mg/dL) compared to normal thiamine participants (27.81±6.88 mg/dL) (p = 0.024). The total protein (6.99±0.71 g/dL) (p = 0.037) and albumin (3.89±0.63 g/dL) (p = 0.029) were also significantly lower in the low-thiamine group. The mean random blood sugar of study participants was 95.30±21.40 mg/dL, while the low-thiamine group had low random blood sugar (93.80±18.07 mg/dL) compared to women with normal thiamine (97.74±25.77 mg/dL) (p = 0.047). The mean haemoglobin was also lower in the low-thiamine group (8.81±1.40 g/dL) compared to the normal thiamine group (10.22±1.34 g/dL) (p = 0.038). The red blood cell count was again lower in the low-thiamine group (3.87±0.55*10^3^/mL) compared to the normal thiamine group (4.12±0.59*10^3^/mL) (p = 0.028).

The majority of participants were gravida first at presentation belonging to the third trimester of pregnancy (68.5%) (Table 2). There was a higher proportion of low-thiamine women who experienced nausea [82.1% vs 69.9% (p = 0.047)] and vomiting [73.6% vs 61.6% (p = 0.05)] compared to participants with normal thiamine. Women with low thiamine had a significant history of loss of appetite [70.5% vs 53.5% (p = 0.036)], lethargy [17.5% vs 9.5% (p = 0.001)] and compared to participants with normal thiamine. Compared to normal thiamine low thiamine status was associated with increased heart rate >100 bpm [6.2% vs 14.3% (p = 0.032)], and PND/Orthopnoea [2.3% vs 9.1% (p = 0.042)]. Anaemia (defined as Hb < 10.9 g/dl [29]) was significantly associated with low thiamine status as against the women with normal thiamine [6.3% vs 1.6% (p = 0.016)].

All the participants consumed rice as a staple diet and there was more number of women with low thiamine status who consumed higher proportions of polished white ration rice (60.6%), compared to locally grown Kashmiri rice (25.1%) (p = 0.004). The rice was customarily washed three times by the majority of women (56.4%) with low thiamine while only two times by most women (36.8%) with normal thiamine status. Low thiamine status of women was significantly associated with the cooking practice of discarding fluids after boiling the rice compared to those not discarding fluids after boiling [72.3% vs 51.3% (p = 0.051)]. The higher consumption of tea, mostly Kashmiri pink tea, was significantly associated with low thiamine status with more women (87.8%) with low thiamine consuming more than two cups of tea daily and only 73.1% with normal thiamine consuming two cups daily (p = 0.071). The consumption of pickles was also higher in the low thiamine group with 48.5% reporting consuming pickles occasionally and 14.9% having them daily. Among women with normal thiamine, occasional pickle intake was reported in 61.5% and daily intake in 5.6% (p = 0.031).

The dietary pattern of the study participants is given in Table 3. The total energy consumption per day was 1421.43±170.11 Kcal/day for women with low thiamine status and 1723.51±87.89 Kcal/day for women with normal thiamine levels (p = 0.024). The total thiamine consumption per day among women with low thiamine status was 0.70±0.15 mg/day and 0.81±0.13 mg/day among women with normal thiamine status (p = 0.048). The average calories derived from proteins were lower in women with low thiamine status (190.12±28.56) compared to those with normal thiamine (219.08±14.72) (p = 0.047). The average calories derived from carbohydrates per day for women with low thiamine status was 920.32±113.48 Kcal while for women with normal thiamine was 1058.32±78.32 Kcal (p = 0.027).

**Table 3:** Dietary analysis of the study population.

Dietary component	Low thiamine Group (n = 188)	Normal thiamine Group (n = 304)		P-value
Protein (gms)	47.53±7.14	54.77±3.68		0.043
Total Kcal from protein	190.12±28.56	219.08±14.72		0.047
Total Fat (gms)	38.75±6.35	41.12±7.05		0.325
Total Kcal from fat	348.75±57.15	370±63.45		0.317
Carbohydrate (gms)	230.08±28.37	264.58±19.58		0.049
Total Kcal from carbohydrate	920.32±113.48	1058.32±78.32		0.027
**Total Energy (Kcal)**	**1421.43±170.11**	**1723.51±87.89**		**0.024**
Total Dietary Fibre (gms)	24.44±5.32	25.51±5.83		0.594
**Micronutrients**				
Thiamine (mgs)	0.70±0.15	0.81±0.13		0.048
Riboflavin (mgs)	0.71±0.13	0.74±0.08		0.081
Calcium (mgs)	549.30±112.83	559±117.60		0.799
Iron (mgs)	7.95±1.75	9.86±1.11		0.048
**Food groups**				
Cereals and Millets (g/d)	248.60±38.8	286.17±27.96		0.046
Cooked rice (g/d)	466±28.3	513±12.07		0.054
Green Leafy Vegetables (g/d)	67.53±22.98	81.81±35.62		0.017
Fruits (g/d)	54.35±4.41	33.49±5.67		0.028
Roots and Tubers (g/d)	35.63±23.91	59.61±32.44		0.044
Milk and Milk Products (g/d)	282.66±97.69	251.32±93.75		0.373
Egg and Egg Products (g/d)	19.12±1.93	35.39±1.76		0.023
Poultry (g/d)	9.39±3.69	9.80±1.54		0.635
Animal Meat (g/d)	10.12±5.01	14.42±4.30		0.037
Edible Oils and Fats (g/d)	17.89±6.09	17.47±3.82		0.807
**Percentage of Energy derived from**				
	**Low thiamine Group (n = 188)**		**Normal thiamine Group (n = 304)**	
Total Kcal from protein	13.37%		12.71%	
Total Kcal from fat	24.53%		21.46%	
Total Kcal from carbohydrate	64.74%		61.40%	

Among women with low thiamine, 3.1% (n = 6) reported perinatal mortality while only 0.3% (n = 1) in the normal thiamine group reported perinatal mortality (p<0.05). Among six infants, three had respiratory distress and needed resuscitation at birth, two had developed shock within two days of birth and one reportedly had no apparent cause, but the mothers reported vague symptoms like refusal to breastfeed, vomiting, and persistent crying. Additionally, low thiamine was associated with poor perinatal outcomes, with more infants needing hospital admissions at birth [11.4% vs 2.8% (p = 0.043)], and early resuscitation [4.7% vs 1.2% p = 0.023] compared to the infants whose mothers had normal thiamine levels (Table 4).

**Table 4:** Perinatal outcome of the study population.

Outcome	All Participants (n = 492)	Low thiamine Group (n = 188)	Normal thiamine Group (n = 304)	P-value
**Perinatal outcome**				
Death	7(1.4)	6 (3.1)	1 (0.3)	0.014
Early neonatal hospital admissions	31 (6.2)	22 (11.5)	9 (2.8)	0.043
Early resuscitation	13 (2.5)	9 (4.7)	4 (1.2)	0.023

## Discussion

This study is the first representative data on thiamine status in pregnant women attending the antenatal clinic in a northern state of India with endemic thiamine deficiency. Our study found a high prevalence of poor thiamine status among pregnant women with 38.2% (n = 188) participants reporting low thiamine levels. This result was consistent with the study conducted by Whitfield and colleagues on Cambodian women of childbearing age wherein they reported a prevalence of 27% using the most conservative cut-off of thiamine <120 nmol/L [30]. Another study conducted by Bourassa and coworkers in Gambian women of reproductive age reported that 35.8% of women were at high-risk ETKac ≥1.25. There are no studies available to determine the thiamine demand of a developing fetus or exclusively breastfed infants and the cutoff level for whole-blood thiamine during pregnancy has not been established [31]. There are several reports of thiamine deficiency from our area with varied manifestations including an infantile form [15–18], Wernicke’s encephalopathy [32] in adults, postpartum polyneuropathy [23], a gastric form of beriberi [33] and high-output heart failure of young [34]. Given the number of cases of infantile beriberi from Kashmir combined with the clinical evidence of thiamine deficiency in peripartum women, we expect that thiamine deficiency during pregnancy is under-recognized and underreported due to varied manifestations in addition to the possibility of subclinical deficiency. No studies so far have reported the burden of thiamine deficiency in pregnant women in our population. In the current study, we have attempted to present the clinical and laboratory gamut of thiamine deficiency during pregnancy and its association with perinatal outcomes.

Infant mortality due to beriberi still remains a problem across different communities worldwide [35–37]. After delivery, the infants grow rapidly and the demand for thiamine rises steeply while the breastmilk of a thiamine-deficient mother may not contain thiamine levels commensurate with these growing demands of the infant [38]. Few studies have reported the sequestration of thiamine in utero keeping thiamine levels normal in newborns and making neonatal deficiency rare [39,40] additionally, previous studies suggest that thiamine deficiency peaks at 3 months of age due to changes in metabolic activity [41]. On contrary, our study reports early infant mortality and poor perinatal outcome in association with the low thiamine status of the mother, suggesting a possibility of thiamine deficiency in the early neonatal period. This was in consonance with the previous studies reporting either infantile beriberi or sudden infant death syndrome (SIDS) among infants born to thiamine-deficient mothers. Jeffrey et al. [42] reported a high incidence of thiamine deficiency in ‘near-miss’ sudden infant death syndrome (SIDS) infants and their mothers and in siblings of SIDS from a westernized Caucasian community in Australia. The thiamine-deficient infants had a high familial incidence of SIDS deaths. These ‘high-risk families might reflect poor nutrition or genetic defects of thiamine uptake and metabolism. Another study conducted by Barennes et al. [10], reported that infantile beriberi in northern Laos mainly occurred in infants breastfed by mothers with an inadequate intake of thiamine. They reported 5.6% of infant deaths while 90.2% of infants recovered after receiving a prompt treatment of parenteral thiamine.

**Fig 1:**
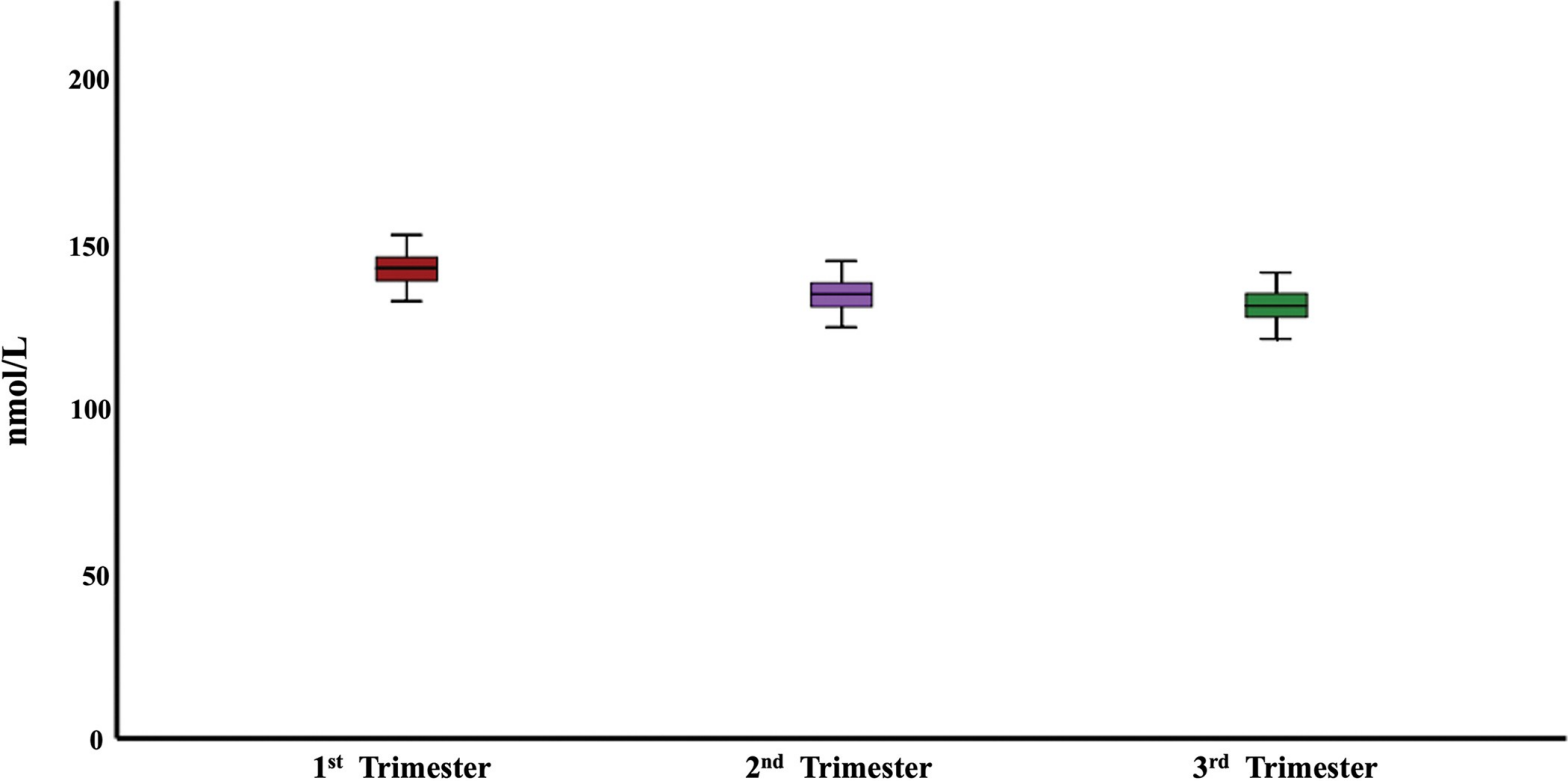
Thiamine levels (nmol/L) during three trimesters of pregnancy.

A fall in whole-blood thiamine with the advance in pregnancy across three trimesters has been noted in our study, however, the finding did not reach statistical significance (Fig 1). This is consistent with the increased physiological demands during pregnancy. In pregnancy, the renal plasma flow and glomerular filtration rate (GFR) both increase, compared to non-pregnant levels, by 40–65% and 50–85%, respectively [43] which could possibly lead to increased excretion of thiamine. Although previous studies indicate the preferential delivery of thiamine to the developing fetus during pregnancy [9] hence, given that we only have maternal thiamine status, it is possible that lower thiamine level in the third trimester is simply a signal of increasing demand from growing fetus with the progression of pregnancy.

We reported a significant association between low thiamine status and lower socioeconomic status. This observation is consistent with a study in which mothers of infants with clinical beriberi were poorer and had received less formal education than the mothers of the control infants [44]. Thiamine deficiency has primarily been reported in Africa, and Asia with the majority of cases from Southeast Asia. This has predominantly been attributed to the disparities in the nutrient content in the general food supply, dietary habits including relying on low-thiamine staple foods, and avoidance of certain foods [45]. Also, people living in refugee camps, prison populations, and those living in extremely poor conditions are more likely to experience a nutritional shortage, especially of water-soluble nutrients [46,47]. Nonetheless, poor socioeconomic status can itself be an independent risk factor for low thiamine status.

In our study population, low thiamine was significantly associated with higher consumption of white-polished ration rice. The washing of rice more than three times was also associated with low thiamine levels in whole blood. A high carbohydrate white-milled polished rice is the staple food of Kashmir consumed twice daily and customarily washed 2–3 times before cooking and the cooking water is discarded after boiling. This cooking practice along with polishing deprives the rice of thiamine rich envelop [48]. Additionally, culturally determined prenatal and postpartum dietary restrictions (eating mostly white rice and soup) and food avoidances (pulses and cheese) [49] provide insufficient thiamine. This along with the rapid depletion of the body’s stores of thiamine within 18 days of a deficient state [50] likely add to the problem. We observed that the higher the consumption of Kashmiri salt pink tea (specially prepared by boiling over long periods of time with baking soda) and pickles, the lesser the thiamine content in the blood. This again is in consonance with the previous data that reports the presence of thiamine antagonists and thiaminases in various foods like pickles and tea, leading to insufficiency of thiamine in adults [31,51]. Also, the process of preparation of pickles includes the fermentation step which causes loss of thiamine due to leaching into the fermentation brine, and the same has been found in the packing step [52]. The percentage of calories derived from carbohydrates is higher in women with low thiamine status. This is consistent with the study conducted by Elmadfa et al. that reports the drop in thiamine levels in the blood and urine as carbohydrate consumption rises from 55% to 65% and 75% of total caloric intake, respectively, for four days in a row [53]. It is known that a high carbohydrate diet increases the requirement of thiamine in the body to a minimum of 0.33 mg per 1,000 Kcal [54].

Thiamine deficiency is the most overlooked condition during pregnancy and breastfeeding due to its variable clinical presentation. Thus, in absence of a readily available test for thiamine detection, the treatment remains a challenging task with clinicians often missing out on simple thiamine challenge to preclude the fatal consequences of thiamine deficiency in the mother as well as in the infant. The present study reports a range of clinical symptoms that were significantly associated with the low thiamine status of women, including, nausea, vomiting, lethargy, loss of appetite, heart rate >100 bpm and PND/Orthopnoea. Many studies have previously reported that the deficiency of thiamine is exacerbated in hyperemesis gravidarum by the decreased absorption produced by intractable vomiting [55,56]. While the demand for thiamine is estimated to increase by >45% during pregnancy [57], loss of appetite leads to inadequate consumption of thiamine-rich foods (such as beef, and eggs) which further causes prodromal characteristics of thiamine deficiency.

The biochemical parameters reveal a significant difference in serum total protein, serum albumin and serum urea between the women with low thiamine status and those with normal thiamine. This difference explains the low nutritional status of pregnant women in Kashmir. Again, the low thiamine group had lower random blood sugar compared to women with normal thiamine which again explains the overall low-calorie intake. Low haemoglobin in association with low thiamine in our population also explains the lower nutritional status of women. This is in line with the study conducted by Ayensu and coworkers who reported poor dietary diversity, undernutrition, and anaemia associated with pregnancy [58]. The biochemical parameters associated with thiamine deficiency have not been explained previously in our population.

Since the frequency of thiamine deficiency and/or suboptimal thiamine status varies greatly depending on the cut-off utilized, assessing the gravity of low thiamine status as a public health concern in Kashmir is challenging. Our study notably is the first large-scale evaluation of pregnant women for whole blood thiamine levels using high-performance liquid chromatography (HPLC). Although the measurement of erythrocyte transketolase activity is the most reliable method for the estimation of thiamine levels, but the assessment of whole-blood thiamine by HPLC has overcome several limitations of the functional assay and exhibits strong correlations with erythrocyte transketolase activity coefficient [19]. This method, used to determine thiamine status, has developed over time. Nevertheless, it is pertinent to mention that the whole-blood ThDP levels in our study were not normalized to RBC volume or haemoglobin concentrations, which substantiates large population-based studies. Our study explores the burden of thiamine deficiency in the vulnerable state of pregnancy because thiamine-deficient mothers put their infants at an increased risk of infantile beriberi [38,39,59] and in turn mortality [60]. However, a possible recall bias while interviewing pregnant women regarding the nature of signs and symptoms and diet remains a major limiting factor in our study. Also, we enrolled the study participants from the hospital setting, which is an important limitation of this study. Further studies are needed to elucidate the full potential of thiamine supplementation in the gravid and breastfeeding states as well as to determine the cut-off levels of whole blood thiamine in these states.

## Conclusion

In conclusion, a high prevalence of biochemical evidence of thiamine deficiency is reported in pregnant women of Kashmir. This has primarily been associated with poor nutritional status. The use of white polished milled ration rice as a staple and intake of foods with possible interference (like tea) with thiamine absorption are among the primary contributory factors of thiamine deficiency in our population. All the more important is that poor thiamine status is associated with poor perinatal outcomes in our study. In addition to the overt thiamine deficiency in infants, there remains a possibility of subclinical thiamine deficiency leading to subtle cognitive and psychomotor defects in infants which needs to be evaluated. Further, it is likely that the initiatives to supplement thiamine in pregnant women and to improve their overall nutritional health would serve a better outcome in terms of infant mortality.
